# Socioeconomic factors and long-term mortality risk after surgical aortic valve replacement

**DOI:** 10.1016/j.ijcrp.2023.200223

**Published:** 2023-11-08

**Authors:** Maria Lachonius, Kok Wai Giang, Martin Lindgren, Kristofer Skoglund, Pétur Pétursson, Martin Silverborn, Anders Jeppsson, Susanne J. Nielsen

**Affiliations:** a Department of Molecular and Clinical Medicine, Sahlgrenska Academy, Gothenburg University, Sweden; b Department of Cardiology, Sahlgrenska University Hospital, Gothenburg, Sweden; c Sahlgrenska University Hospital/Östra, Department of Medicine, Geriatrics and Emergency Medicine/Östra, Gothenburg, Sweden; d Department of Cardiothoracic Surgery, Sahlgrenska University Hospital, Gothenburg, Sweden

**Keywords:** Aortic stenosis, Mortality, Socioeconomic status, Surgical aortic valve replacement

## Abstract

**Background:**

There is scarce knowledge about the association between socioeconomic status and mortality in patients undergoing surgical aortic valve replacement. This study explores the associations between income, education and marital status, and long-term mortality risk.

**Methods:**

In this national registry-based observational cohort study we included all 14,537 patients aged >18 years who underwent isolated surgical aortic valve replacement for aortic stenosis in Sweden 1997–2020. Socioeconomic status and comorbidities were collected from three mandatory national registries. Cox regression models adjusted for patient characteristics and comorbidities were used to estimate the mortality risk.

**Results:**

Mortality risk was higher for patients in the lowest versus the highest income quintile (adjusted hazard ratio [aHR] 1.36, 95 % confidence interval [CI]: 1.11–1.65), for patients with <10 years education versus >12 years (aHR 1.20, 95 % CI:1.08–1.33), and for patients who were not married/cohabiting versus those who were (aHR 1.24, 95 % CI:1.04–1.48). Patients with the most unfavorable socioeconomic status (lowest income, shortest education, never married/cohabiting) had an adjusted median survival of 2.9 years less than patients with the most favorable socioeconomic status (14.6 years, 95 % CI: 13.2–17.4 years vs. 11.7 years, 95 % CI: 9.8–14.4).

**Conclusions:**

Low socioeconomic status in patients undergoing surgical aortic valve replacement is associated with shorter survival and an increased long-term adjusted mortality risk. These results emphasize the importance of identifying surgical aortic valve replacement patients with unfavorable socioeconomic situation and ensure sufficient post-discharge surveillance.

## 1 Introduction

Low socioeconomic status (SES), especially short education, is associated with increased mortality in patients with cardiovascular disease [[1], [2], [3]]. Socioeconomic disadvantages are also associated with cardiovascular risk factors such as diabetes, hypertension, smoking, and physical inactivity [[4], [5], [6], [7]], and with an increased long-term mortality risk and shortened median survival in coronary artery bypass grafting patients [8]. Surgical aortic valve replacement (SAVR) is recommended in younger patients with symptomatic severe aortic stenosis and low surgical risk [9,10]. There is scarce knowledge about the impact of socioeconomic factors on outcome after SAVR [11,12]. Bagger et al. found a moderately increased mortality risk in a mixed population of isolated heart valve surgery patients using a multifactorial socioeconomic scoring system [11], and Dalén et al. found that low income was associated with an increased risk of post-discharge bleeding and mortality in patients receiving a mechanical aortic prosthesis [12].

To our knowledge, there are no previous studies investigating associations between individual socioeconomic factors and mortality in patients with aortic stenosis undergoing SAVR, and no studies investigating if there are sex-specific differences in the associations between socioeconomic factors and mortality after SAVR. The aim of the present study was therefore to explore the association between individual socioeconomic factors and long-term mortality risk in men and women undergoing isolated SAVR due to severe aortic stenosis.

## 2 Material and methods

### 2.1 Study design, study population, and data sources

All patients ≥18 years of age (n = 14,537) who were diagnosed with aortic stenosis and underwent primary isolated SAVR in Sweden from 1997 to 2020 were included in a nationwide observational population-based cohort study. Outcome measure was all-cause mortality. Median follow-up was 7.3 years (interquartile range: 3.9–11.4). The patients were identified from the Swedish Cardiac Surgery Registry, which is a part of the Swedish Web-system for Enhancement and Development of Evidence-based care in Heart disease Evaluated According to Recommended Therapies (SWEDEHEART) registry [13]. The Swedish Cardiac Surgery Registry includes patient characteristics, intraoperative details, and early complications in all open cardiac operations in Sweden since 1992 [14].

Codes for surgical procedures were defined by the Swedish National Board of Health and Welfare (Classification of Surgery, sixth edition, 1987, and Nordic Classification of Surgical Procedures, 1997). To identify patients undergoing a first isolated SAVR, the codes FMD 00 and FMD 10 were used in combination with the diagnostic code for aortic stenosis (I35.0) according to the 10th revision of the International Classification of Diseases (ICD-10). A total of 3946 patients were excluded because of congenital heart disease (n = 623), history of endocarditis (n = 1168), or diagnosis other than aortic stenosis (n = 1532). A flowchart describing included and excluded patients is depicted in Supplemental Figure 1.

Baseline characteristics and comorbidities were obtained from SWEDEHEART and from the Swedish National Patient Register (NPR), where registration is mandatory for all hospitals in Sweden. The NPR has a complete national coverage from 1987 for all diagnoses and all patients hospitalized in Sweden, with an overall diagnosis validity of 85%–95 % for cardiovascular diseases [15]. Diagnoses in the NPR are based on ICD codes, with the 9th revision (ICD-9) used from 1987 to 1997 and the 10th revision (ICD-10) from 1997 to 2015. Data for social variables were collected from the Longitudinal Integration Database for Health Insurance and Labor Market Studies register (LISA). This register is annually updated and includes all citizens in Sweden aged ≥16 years [16].

### 2.2 Socioeconomic status

Marital status was divided into four categories: married/cohabiting, never married, divorced, and widowed. Length of education was stratified into three levels: <10 years (compulsory school only), 10–12 years (upper school), and >12 years (college/university level). Income was measured as annual household disposable income at year of surgery, stratified into five quintiles from Q1 (lowest) to Q5 (highest). The consumer price index according to Statistics Sweden (SCB) was used to adjust for inflation rates over time. If data were missing for the year of surgery, the latest information about education, marital status, and income was imputed from records for the most recent years before the surgery. In total, 164 (1.10 %) individuals had missing data regarding education. There was no missing data for income and marital status. The study population was divided into groups for comparisons between socioeconomic conditions; the most favorable SES group was defined as being married or cohabiting, education >12 years, and highest income level (Q5), and the least favorable SES group was defined as never being married/cohabiting, education <10 years, and lowest income level (Q1).

The Swedish Cause of Death Register, which includes information on all deaths of Swedish citizens [17] was used to obtain death dates. Data from the four national registers were linked together through a personal 10-digit social security number, unique for all Swedish citizens.

### 2.3 Statistical methods

Baseline characteristics are presented as frequencies together with percentages for categorical variables. Continuous variables are presented as means with standard deviations (SD). Follow-up time started at date after discharge, and ended at death or end of study, whichever occurred first. Incidence rates were estimated as total follow-up in the number of deaths divided by the follow-up in years and reported as incidence rate per 100 person-years with 95 % confidence intervals (CI).

Survival probabilities were calculated using Kaplan-Meier survival curves with 95 % CI, and median survival. For all analyses, a p-value of <0.05 was considered statistically significant.

Cox proportional hazards regression was used to calculate the crude (HR) and adjusted (aHR) mortality risk, separately for marital status, education, and income. The multivariate models were adjusted for baseline characteristics (age, body mass index, estimated glomerular filtration sex, smoking, myocardial infarction, diabetes, hypertension, heart failure, left ventricular ejection fraction, atrial fibrillation, previous stroke, chronic respiratory disease, peripheral vascular disease, renal insufficiency, rate history of cancer, rheumatic aortic stenosis, depression, bicuspid aortic valve stenosis, year of surgery, urgency of surgery, type of valve prothesis and for socioeconomic factors other than the response variable (marital status, education, income). All statistical analyses were performed using version 4.2.0 of R [18].

### 2.4 Ethical considerations

The present study was performed in line with the Declaration of Helsinki and was approved by the Swedish Ethical Review Authority (registration number: 2021-00122). All personal identifiers were replaced by codes before analysis to ensure anonymity. The need for individual patient consent was waived by the committee. This article follows the recommendations from the statement of Strengthening the Reporting of Observational Studies In Epidemiology (STROBE) [19].

## 3 Results

### 3.1 General

In total, 14,537 patients (44.6 % women) who underwent SAVR because of aortic stenosis were included (mean age: 69.7, SD: 10.1). At baseline, 51.9 % of the patients had hypertension, 38.0 % atrial fibrillation, 21.2 % heart failure, 23.3 % hyperlipidemia, 17.2 % diabetes, and 53.4 % had a history of smoking (Table 1).

**Table 1:** Baseline characteristics by sex in 14,537 patients who underwent isolated surgical aortic valve surgery.

	Total n (%)	Men n (%)	Women n (%)	*P*-value
Number of patients	14,537	8059 (55.4)	6478 (44.6)	
Mean age (SD)	69.7 (10.1)	67.8 (10.4)	72.1 (9.0)	<0.001
BMI (SD)	27.2 (5.4)	27.1 (4.4)	27.4 (6.5)	0.002
Missing	1351 (9.3)	715 (8.9)	636 (9.8)	0.054
eGFR (SD)	72.3 (18.6)	75.4 (18.2)	68.5 (18.4)	<0.001
Missing	929 (6.4)	475 (5.9)	454 (7.0)	0.007
Comorbidities:				
Ever smoked	5118 (53.4)	3387 (61.1)	1731 (42.8)	<0.001
Myocardial infarction	1111 (7.6)	689 (8.5)	422 (6.5)	<0.001
Diabetes	2507 (17.2)	1466 (18.2)	1041 (16.1)	0.001
Hypertension	7541 (51.9)	4036 (50.1)	3505 (54.1)	<0.001
Heart failure	3076 (21.2)	1744 (21.6)	1332 (20.6)	0.118
Left ejection fraction <50 %	2336 (20.1)	1599 (24.3)	737 (14.6)	<0.001
Missing	2894 (19.9)	1466 18.2)	1428 (22.0)	<0.001
Atrial fibrillation	5523 (38.0)	3054 (37.9)	2469 (38.1)	0.801
Previous stroke	1117 (7.7)	622 (7.7)	495 (7.6)	0.887
Chronic respiratory disease	1621 (11.2)	813 (10.1)	808 (12.5)	<0.001
Peripheral vascular disease	846 (5.8)	502 (6.2)	344 (5.3)	0.021
Renal insufficiency	637 (4.4)	391 (4.9)	246 (3.8)	0.002
History of cancer	2302 (15.8)	1255 (15.6)	1047 (16.2)	0.345
Hyperlipidemia	3381 (23.3)	1929 (23.9)	1452 (22.4)	0.032
Rheumatic aortic valve stenosis	524 (3.6)	267 (3.3)	257 (4.0)	0.04
Depression	526 (3.6)	261 (3.2)	265 (4.1)	0.007
Bicuspid aortic valve stenosis	191 (1.3)	131 (1.6)	60 (0.9)	<0.001
Mechanical prosthesis valve	3802 (26.2)	2429 (30.1)	1373 (21.2)	<0.001
Marital status:				<0.001
Married or cohabiting	8192 (56.4)	5124 (63.6)	3068 (47.4)	
Never married	1711 (11.8)	1188 (14.7)	523 (8.1)	
Divorced	2105 (14.5)	1177 (14.6)	928 (14.3)	
Widowed	2529 (17.4)	570 (7.1)	1959 (30.2)	
Education:				
<10 years	6081 (41.8)	3153 (39.1)	2928 (45.2)	<0.001
10–12 years	5523 (38.0)	3185 (39.5)	2338 (36.1)	
>12 years	2769 (19.0)	1641 (20.4)	1128 (17.4)	
Income:				
Q1 (lowest)	2908 (20.0)	972 (12.1)	1936 (29.9)	<0.001
Q2	2908 (20.0)	1442 (17.9)	1446 (22.6)	
Q3	2907 (20.0)	1695 (21.0)	1212 (18.7)	
Q4	2907 (20.0)	1867 (23.2)	1040 (16.1)	
Q5	2907 (20.0)	2083 (25.8)	824 (12.7)	
Socioeconomic status:				<0.001
Highest	1417 (9.8)	958 (11.9)	459 (7.1)	
Lowest	469 (3.2)	328 (4.1)	141 (2.2)	
Other	12,617 (87.0)	6750 (84.0)	5867 (90.7)	

BMI = Body mass Index, eGFR = Estimated glomerular filtration rate, Q = Quintile, SD = Standard deviation.

Supplemental tables 2-4 shows baselines characteristics, stratified by socioeconomic variables. Patients with low income and short education were generally older and had more comorbidities, and underwent less often mechanical prosthesis implantation, than patients with higher income or longer education.

### 3.2 Survival

**Fig. 1:**
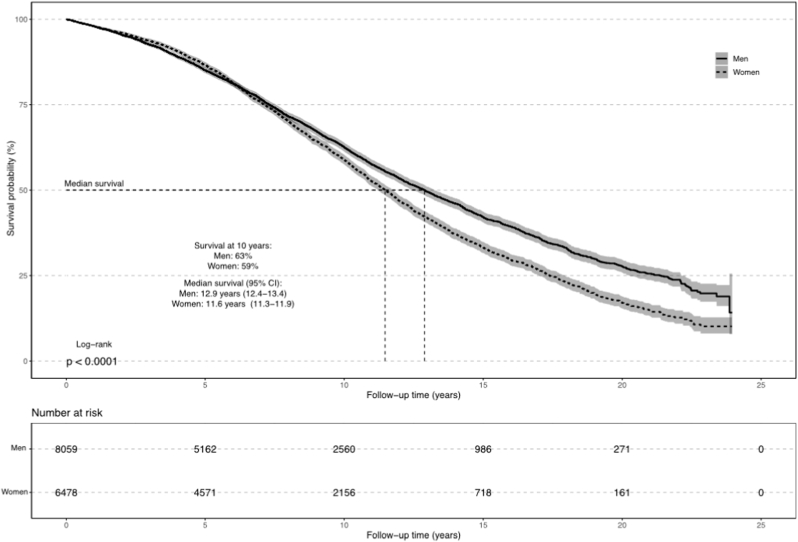
Unadjusted survival in patients who underwent isolated surgical aortic valve surgery.

In total, 6202 (42.7 %) of the patients died during the follow-up (Supplemental figure 5). Ten years after SAVR, the overall survival was 63 % among men and 59 % among women. The unadjusted median survival was 12.9 years in men (95 % CI:12.4–13.4) and 11.6 years (95 % CI:11.3–11.9) in women (Fig. 1). Corresponding results for adjusted median survival was 11.5 years (95 % CI: 11.0–12.1) in men and 13.0 years (95 % CI: 12.3–14.2) in women (Supplemental figure 2). The overall incidence rate of mortality was 5.27 (95 % CI: 5.14–5.40)/100 person years (Supplemental table 6). In total 509 (3.5 %) patients underwent reintervention during the follow up.

### 3.3 Income

**Fig. 2:**
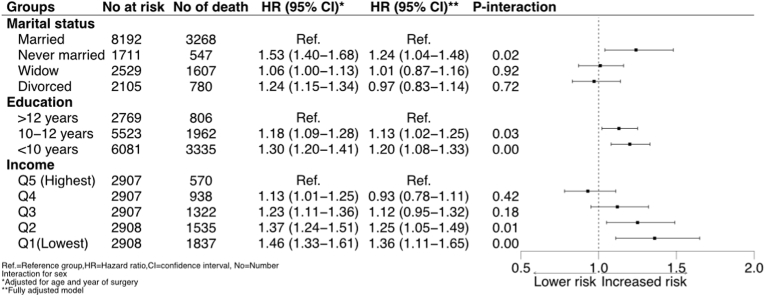
Multivariable adjusted hazard ratio for all-cause mortality according to marital status, education, and income in patients who underwent isolated surgical aortic valve surgery.

Hazard ratio adjusted for age, sex and year of surgery showed that patients with the lowest income level had an associated increased risk of mortality during follow-up (HR 1.46, 95 % CI: 1.33–1.61) compared to patients with the highest income level. This associated risk among patients with lowest income level decreased when comorbidities and educational level and marital status were included, but the risk was still significantly associated with an increased risk compared to patient with the highest income level (aHR1.36, 95 % CI: 1.11–1.65) (Fig. 2).

Sex specific analysis showed that men with the lowest income level had an increased risk compared to men with the highest income level (aHR 1.61, 95 % CI: 1.24–2.09). This associated risk was not observed in women (aHR 1.17, 95 % CI: 0.86–1.59). (Supplemental figure 3).

### 3.4 Education

Patients with the lowest education had an associated increased risk of mortality (<10 years; HR 1.30, 95 % CI: 1.20–1.41 and 10–12 years; 1.18, 95 % CI: 1.09–1.28). This associated risk remained in the adjusted analysis (<10 years; aHR 1.20, 95 % CI: 1.08–1.33 and 10–12 years; aHR 1.13, 95 % CI: 1.02–1.25) (Fig. 2).

Among men, neither educational level <10 years nor educational level 10–12 years was associated with an increased risk of mortality (aHR 0.99, 95 % CI: 0.82–1.19; and aHR 1.02, 95 % CI: 0.85–1.22, respectively). In women, both educational level <10 years and educational level 10–12 years were associated with an increased risk of mortality (aHR 1.37, 95 % CI: 1.10–1.71; and aHR 1.30, 95 % CI:1.04−1.63, respectively) (Supplemental Figure 3).

### 3.5 Marital status

Never being married was associated with a higher mortality risk in both unadjusted model (HR 1.53, 95 % CI: 1.40–1.68) and after adjustments for comorbidities, income, and educational level (aHR 1.24, 95 % CI: 1.04–1.48). Among divorced and widowed patients, the associated increased risk in unadjusted models was attenuated in the multi-adjusted HR models (aHR 0.97, 95 % CI: 0.83–1.14) and aHR 1.01, 95 % CI: 0.87–1.16) (Fig. 2). No increased risk was observed among men and women in the separate analysis. (Supplemental Figure 3).

### 3.6 Survival according to the most favorable versus least favorable socioeconomic status

**Fig. 3:**
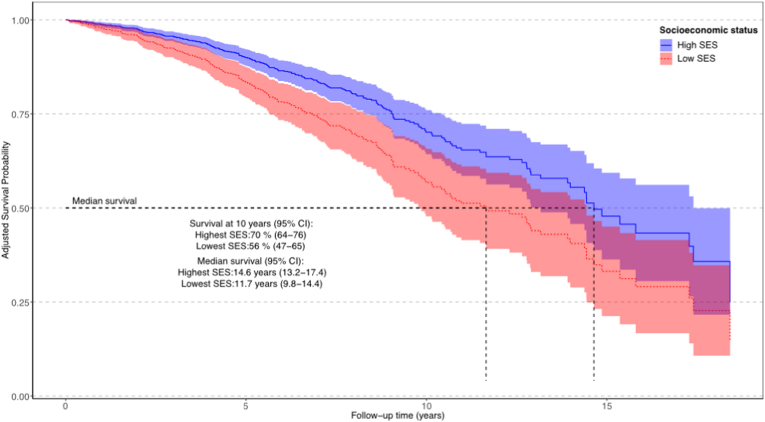
Adjusted survival according to high socioeconomic status versus low socioeconomic status in patients.

The proportion of survival at 10 years was 70 % (95 % CI: 64–76) in patients with the most favorable SES (highest education, married, and highest income level) compared to 56 % (95 % CI: 47–65) in patients with the least favorable SES (lowest education, never married, and lowest income level). Adjusted median survival was 2.9 years shorter in patients with the least favorable SES compared to those with the most favorable SES: 14.6 years (95 % CI: 13.2–17.4) years vs. 11.7 years (95 % CI: 9.8 –14.4) (Fig. 3).

Unadjusted median survival curves are shown in Supplemental figure 4.

## 4 Discussion

In this population-based cohort study of 14,537 individuals, the main finding was that low income, short education, and never been married were associated with shorter survival and higher adjusted mortality risk after SAVR. Patients with the least favorable socioeconomic factors (never married or being married/cohabiting, education <10 years, and lowest income level), had nearly 3 years shorter adjusted median survival after SAVR, than patients with the most favorable socioeconomic status.

To our knowledge, few previous studies have investigated the association between SES and mortality in SAVR patients. Our results demonstrate that disparities in SES conditions are associated with an increased mortality risk after SAVR and is most likely due to multiple factors. Life expectancy after SAVR has also been reported in previous studies. Glaser et al. found that SAVR patients had a higher loss of life compared to the general population [20]. Martinsson et al. showed that median survival was affected by age and the presence of surgical risk factors in SAVR patients receiving a bioprosthesis [21]. In line with the present study, Nielsen et al. [8] found that patients with unfavorable SES who underwent coronary artery bypass grafting lost 4–5 years in median survival compared to patients with favorable SES, where men with low income had a stronger association for mortality than women. This disadvantageous association for risk of mortality in men compared to women with low income, was also observed in the present study.

It is a well-known association between low SES and increased mortality in patients with cardiovascular disease [[1], [2], [3]]. In the present study we could observe that a large proportion of the patients had cardiovascular risk factors such as hypertension, heart failure, atrial fibrillation, and hyperlipidemia at the time of surgery. Although comorbidities at the time of surgery were taken into consideration in the multi-adjusted regression models, several of the diseases related to cardiovascular disease are progressive in a lifetime perspective.

Several previous investigations of educational and economic factors after cardiac surgery have demonstrated an increased mortality risk in patients with low educational and income level [8,11]. This is in line with our finding which may suggest that low educational level have an unfavorable impact on patients' health literacy, which affects information recall and health-related knowledge [22]. Low health literacy leads to less beneficial health behavior regarding exercise, smoking, and medication use [23]. In the present study, about 50 % of the patients had a history of smoking before surgery. Prior studies suggests that cardiovascular- and socioeconomic risk factors, should be evaluated preoperatively, by the Heart Team in dialogue with the patients [9,10,24]. Identifying patients with low SES and evaluate which resources the patients’ needs, may also increase the possibilities to strengthen the health literacy [25,26]. It is therefore important to provide customized and robust advice to vulnerable patients with low SES, in order to increase the possibilities for the patients to understand the importance of adhering to the recommended treatments and a healthy lifestyle after SAVR. Educational level, cultural influence, and financial strain affect the circumstances to adopt a healthy lifestyle [5,[27], [28], [29]].

In the present study, no information of the study population's living areas was available. However, previous studies have shown that living in deprived areas is associated with an increased presence of cardiovascular risk factors [5,[27], [28], [29]]. Exploring the association with patient's living area and mortality risk after SAVR entitle further studies.

Recommended secondary prevention medications are less often dispensed to patients who undergoes coronary artery bypass grafting with low income [30]. There is a scarce knowledge about the association between low SES and the use of secondary prevention medication and mortality risk in SAVR patients. However, two recent studies have showed that statins and RAAS inhibitors were associated with reduced mortality after SAVR [31,32]. It is possible that low income affects the dispensation of important secondary prevention medication also in SAVR patients. In Sweden, the patients are covered by a general health care insurance which contribute to a protection for high medication costs where the patients pay the first 2600 SEK (∼240 $) for medical prescriptions within a year, and the first 1300 SEK (∼120 $) for in hospital care, follow up by specialist physician and contact with primary care. Still, for patients with low income this cost may be difficult to manage. This association is not clear, and further studies are warranted, exploring the association of low SES, adherence to prescribed medication and mortality risk after SAVR.

The association between marital status and mortality risk as found in the present study has previously been described with a focus on patients with cardiovascular disease [33] and patients undergoing coronary artery bypass grafting [8,34], but has not previously been reported in SAVR patients. Lack of social support, which is associated with adverse outcome and adverse health-related quality of life after cardiac surgery [35] may be one explanation. SES is a complex variable with multiple components. The result from present study analyzing different factors of SES in SAVR patients. Further studies are needed to examine if availability to medical and social support attenuates the increased long-term risk of mortality after SAVR in patients with low SES.

Strengths of this study include the large population-based cohort, high-quality registry data, and a long and complete follow-up. However, we did not have any information regarding either ethnicity, medication during the follow-up period or adherence to non-medical secondary prevention recommendations such as diet, physical activity, or smoking cessation. Another limitation is that the retrospective study design carries an inherent risk of selection bias and residual confounding.

## 5 Conclusion

In conclusion, low SES is associated with an increased adjusted mortality risk after SAVR. These results emphasize the importance of identifying SAVR patients with unfavorable socioeconomic status and ensure sufficient post-discharge surveillance.

## Credit author statement

Maria Lachonius: Formal analysis, Investigation, Project administration, Writing – original draft, Writing – review & editing.Susanne J. Nielsen: Conceptualization, Data curation, Formal analysis, Investigation Methodology, Project administration, Funding acquisition, Supervision, Validation, Writing – original draft, Writing – review & editing.Kok Wai Giang: Data curation, Formal analysis, Investigation, Methodology, Software, Supervision, Validation, Visualization, Writing – review & editing.Anders Jeppsson: Methodology, Funding acquisition, Supervision, Writing – original draft, Writing – review & editing.Kristofer Skoglund: Supervision, Writing – review & editing.Pétur Pétursson: Supervision, Writing – review & editing.Martin Lindgren: Funding acquisition, Writing – review & editing.Martin Silverborn: Writing – review & editing

## Sources of funding

The study was supported by the Swedish state under the agreement between the Swedish government and the county councils concerning economic support of research and education of doctors (ALF agreement; grant no ALFGBG-971608 to MLi and ALFGBG-942665 to SN) and the Nils Winberg Family Foundation (grant to AJ).

## Declaration of competing interest

AJ has received fees for consultancy from 10.13039/100020948AstraZeneca, Werfen and LFB Biotechnologies outside the present work. None of the other authors have anything to disclose.
