# Supplementary material for: Socioeconomic factors and long-term mortality risk after surgical aortic valve replacement
Source: Int J Cardiol Cardiovasc Risk Prev. 2023 Nov 8;19:200223. doi: 10.1016/j.ijcrp.2023.200223 (PMC10661603; doi:10.1016/j.ijcrp.2023.200223)
Supplement: Multimedia component 1 [file mmc1.docx]

**SUPPLEMENTAL MATERIAL**

**Socioeconomic factors and long-term mortality risk after surgical aortic valve replacement**

Maria Lachonius, Kok Wai Giang, Martin Lindgren, Kristofer Skoglund, Pétur Pétursson, Martin Silverborn, Anders Jeppsson, Susanne J. Nielsen.

**Supplemental table 1**. ICD codes used for surgical aortic valve replacement patients.

| **Diagnosis** | **ICD 9** | **ICD 10** |
| --- | --- | --- |
| Myocardial infarction | 410 | I21 |
| Diabetes | 250 | E10–E14 |
| Hypertension | 401–405 | I10–I15 |
| Heart failure | 427.00,427.10 | I50 |
| Atrial fibrillation | 427D | I48 |
| Stroke | 431–434, 436; | I61–I64, I69 |
| Chronic respiratory disease | 490–496 | J40–J47 |
| Peripheral vascular disease | 440, 441, 442, 443, 444, 447 | I65–I65.9, I70–174, I77 |
| Renal insufficiency | 584–586 | N17–N19 |
| Malignancy | 140–208 | C00–C97 |
| Rheumatic aortic valve stenosis | 395A | I06.0 |

**Supplemental table 2.** Baseline characteristics by income level in 14 537 patients who underwent isolated surgical aortic valve surgery.

|  | **Total** | **Income** | | | | |
| --- | --- | --- | --- | --- | --- | --- |
|  | **n (%)** | **Q1 (lowest)**  **n (%)** | **Q2**  **n (%)** | **Q3**  **n (%)** | **Q4**  **n (%)** | **Q5 (highest)**  **n (%)** |
| **Number of patients** | 14537 | 2908 (20.0) | 2908 (20.0) | 2907 (20.0) | 2907 (20.0) | 2907 (20.0) |
| Mean age (SD) | 69.7 (10.1) | 73.4 (9.3) | 72.2 (9.3) | 71.2 (9.2) | 67.7 (9.9) | 64.3 (9.7) |
| BMI (SD) | 27.2 (5.4) | 27.2 (5.8) | 27.2 (6.2) | 27.1 (4.7) | 27.4 (5.6) | 27.3 (4.7) |
| eGFR (SD) | 72.3 (18.6) | 67.0 (18.8) | 69.4 (18.3) | 71.4 (18.2) | 74.4 (18.2) | 79.0 (17.0) |
| **Comorbidities** |  |  |  |  |  |  |
| Ever smoked | 5118 (53.4) | 765 (46.9) | 947 (53.6) | 1025 (53.4) | 1174 (58.5) | 1207 (53.5) |
| Myocardial infarction | 1111 (7.6) | 242 (8.3) | 269 (9.3) | 217 (7.5) | 214 (7.4) | 169 (5.8) |
| Diabetes | 2507 (17.2) | 534 (18.4) | 560 (19.3) | 501 (17.2) | 495 (17.0) | 417 (14.3) |
| Hypertension | 7541 (51.9) | 1446 (49.7) | 1518 (52.2) | 1549 (53.3) | 1536 (52.8) | 1492 (51.3) |
| Heart failure | 3076 (21.2) | 814 (28.0) | 701 (24.1) | 620 (21.3) | 520 (17.9) | 421 (14.5) |
| Left ejection fraction < 50 | 2336 (20.1) | 446 (21.9) | 478 (21.9) | 492 (20.6) | 441 (18.5) | 479 (18.1) |
| Atrial fibrillation | 5523 (38.0) | 1183 (40.7) | 1180 (40.6) | 1158 (39.8) | 1047 (36.0) | 955 (32.9) |
| Previous stroke | 1117 (7.7) | 250 (8.6) | 261 (9.0) | 237 (8.2) | 212 (7.3) | 157 (5.4) |
| Chronic respiratory disease | 1621 (11.2) | 361 (12.4) | 332 (11.4) | 348 (12.0) | 315 (10.8) | 265 (9.1) |
| Peripheral vascular disease | 846 (5.8) | 160 (5.5) | 194 (6.7) | 189 (6.5) | 160 (5.5) | 143 (4.9) |
| Renal insufficiency | 637 (4.4) | 147 (5.1) | 114 (3.9) | 148 (5.1) | 135 (4.6) | 93 (3.2) |
| History of cancer | 2302 (15.8) | 399 (13.7) | 495 (17.0) | 490 (16.9) | 490 (16.9) | 428 (14.7) |
| Hyperlipidemia | 3381 (23.3) | 510 (17.5) | 595 (20.5) | 737 (25.4) | 752 (25.9) | 787 (27.1) |
| Rheumatic aortic valve stenosis | 524 (3.6) | 144 (5.0) | 110 (3.8) | 95 (3.3) | 89 (3.1) | 86 (3.0) |
| Bicuspid aortic valve stenosis | 191 (1.3) | 16 (0.6) | 15 (0.5) | 19 (0.7) | 50 (1.7) | 91 (3.1) |
| Mechanical valve prosthesis | 3802 (26.2) | 555 (19.1) | 603 (20.7) | 659 (22.7) | 920 (31.6) | 1065 (36.6) |

BMI = Body mass Index, eGFR = Estimated glomerular filtration rate, Q = Quintile, SD = Standard deviation.

**Table 3.** Baseline characteristics by educational level in 14 537 patients who underwent isolated surgical aortic valve surgery.

|  | **Total** | **Education** | | |
| --- | --- | --- | --- | --- |
|  | **n (%)** | **<10 y**  **n (%)** | **10–12 y**  **n (%)** | **>12 y**  **n (%)** |
| **Number of patients** | 14537 | 6081 (41.8) | 5523 (38.0) | 2769 (19.0) |
| Mean age (SD) | 69.7 (10.1) | 72.0 (8.9) | 68.1 (10.5) | 67.7 (10.4) |
| BMI (SD) | 27.2 (5.4) | 27.3 (4.7) | 27.5 (5.7) | 26.7 (6.4) |
| eGFR (SD) | 72.3 (18.6) | 69.2 (18.4) | 74.3 (18.7) | 75.5 (17.6) |
| **Comorbidities:** |  |  |  |  |
| Ever smoked | 5118 (53.4) | 1922 (52.9) | 2193 (56.6) | 956 (48.3) |
| Myocardial infarction | 1111 (7.6) | 510 (8.4) | 427 (7.7) | 160 (5.8) |
| Diabetes | 2507 (17.2) | 1109 (18.2) | 973 (17.6) | 379 (13.7) |
| Hypertension | 7541 (51.9) | 3131 (51.5) | 2964 (53.7) | 1366 (49.3) |
| Heart failure | 3076 (21.2) | 1513 (24.9) | 1087 (19.7) | 427 (15.4) |
| Left ejection fraction < 50 | 2336 (20.1) | 981 (22.0) | 898 (19.3) | 429 (17.8) |
| Atrial fibrillation | 5523 (38.0) | 2384 (39.2) | 2054 (37.2) | 1022 (36.9) |
| Previous stroke | 1117 (7.7) | 175 (6.3) | 388 (7.0) | 175 (6.3) |
| Chronic respiratory disease | 1621 (11.2) | 676 (11.1) | 664 (12.0) | 263 (9.5) |
| Peripheral vascular disease | 846 (5.8) | 369 (6.1) | 328 (5.9) | 140 (5.1) |
| Renal insufficiency | 637 (4.4) | 267 (4.4) | 254 (4.6) | 105 (3.8) |
| History of cancer | 2302 (15.8) | 940 (15.5) | 866 (15.7) | 476 (17.2) |
| Hyperlipidemia | 3381 (23.3) | 1275 (21.0) | 1431 (25.9) | 637 (23.0) |
| Rheumatic aortic valve stenosis | 524 (3.6) | 239 (3.9) | 173 (3.1) | 98 (3.5) |
| Depression | 526 (3.6) | 205 (3.4) | 215 (3.9) | 101 (3.6) |
| Bicuspid aortic valve stenosis | 191 (1.3) | 34 (0.6) | 85 (1.5) | 71 (2.6) |
| Mechanical valve prosthesis | 3802 (26.2) | 1366 (22.5) | 1617 (29.3) | 790 (28.5) |

BMI = Body mass Index, eGFR = Estimated glomerular filtration rate, Q = Quintile, SD = Standard deviation.

**Table 4.** Baseline characteristics by marital status in 14 537 patients who underwent isolated surgical aortic valve surgery.

|  | **Total** | **Marital status** | | | |
| --- | --- | --- | --- | --- | --- |
|  | **n (%)** | **Married/ cohabit**  **n (%)** | **Never married**  **n (%)** | **Divorced**  **n (%)** | **Widowed**  **n (%)** |
| **Number of patients** | 14537 | 8192 (56.4) | 1711 (11.8) | 2105 (14.5) | 2529 (17.4) |
| Mean age (SD) | 69.7 (10.1) | 69.5 (9.4) | 62.5 (11.9) | 68.0 (9.2) | 76.9 (6.3) |
| BMI (SD) | 27.2 (5.4) | 27.2 (5.7) | 27.4 (5.2) | 27.5 (4.9) | 27.0 (4.8) |
| eGFR (SD) | 72.3 (18.6) | 72.6 (18.0) | 79.4 (20.1) | 74.6 (18.1) | 64.7(17.1) |
| **Comorbidities:** |  |  |  |  |  |
| Ever smoked | 5118 (53.4) | 2924 (53.9) | 654 (54.0) | 935 (62.3) | 605 (41.8) |
| Myocardial infarction | 1111 (7.6) | 609 (7.4) | 115 (6.7) | 165 (7.8) | 222 (8.8) |
| Diabetes | 2507 (17.2) | 1376 (16.8) | 317 (18.5) | 387 (18.4) | 427 (16.9) |
| Hypertension | 7541 (51.9) | 4244 (51.8) | 803 (46.9) | 1094 (52.0) | 1400 (55.4) |
| Heart failure | 3076 (21.2) | 1584 (19.3) | 383 (22.4) | 451 (21.4) | 658 (26.0) |
| Left ejection fraction < 50 | 2336 (20.1) | 1271 (19.4) | 349 (24.4) | 354 (20.1) | 362 (19.1) |
| Atrial fibrillation | 5523 (38.0) | 3022 (36.9) | 529 (30.9) | 814 (38.7) | 1158 (45.8) |
| Previous stroke | 1117 (7.7) | 620 (7.6) | 109 (6.4) | 168 (8.0) | 220 (8.7) |
| Chronic respiratory disease | 1621 (11.2) | 867 (10.6) | 175 (10.2) | 282 (13.4) | 297 (11.7) |
| Peripheral vascular disease | 846 (5.8) | 460 (5 .6) | 92 (5.4) | 143 (6.8) | 151 (6.0) |
| Renal insufficiency | 637 (4.4) | 334 (4.1) | 84 (4.9) | 103 (4.9) | 116 (4.6) |
| History of cancer | 2302 (15.8) | 1368 (16.7) | 200 (11.7) | 311 (14.8) | 423 (16.7) |
| Hyperlipidemia | 3381 (23.3) | 2016 (24.6) | 331 (19.3) | 514 (24.4) | 520 (20.6) |
| Rheumatic aortic valve stenosis | 524 (3.6) | 275 (3.4) | 52 (3.0) | 84 (4.0) | 113 (4.5) |
| Depression | 526 (3.6) | 221 (2.7) | 92 (5.4) | 136 (6.5) | 77 (3.0) |
| Bicuspid aortic valve stenosis | 191 (1.3) | 103 (1.3) | 52 (3.0) | 29 (1.4) | 7 (0.3) |
| Mechanical valve prosthesis | 3802 (26.2) | 2234 (27.3) | 701 (41.0) | 582 (27.6) | 285 (11.3) |

BMI = Body mass Index, eGFR = Estimated glomerular filtration rate, Q = Quintile, SD = Standard deviation.

| Supplemental table 5. Mortality in 14 537 patients who underwent isolated surgical aortic valve surgery. | | | | |
| --- | --- | --- | --- | --- |
|  | **Total**  **n (%)** | **Men**  **n (%)** | **Women**  **N (%)** | **P-value** |
| Number of patients | 14 537 | 8059 (55.4) | 6478 (44.6) |  |
| Mortality: |  |  |  |  |
| All-cause | 6202 (42.7) | 3120 (38.7) | 3082 (47.6) | <0.001 |
| Cardiovascular disease | 2951 (20.3) | 1420 (17.6) | 1531 (23.6) | < 0.001 |
| Malignancy | 1156 (8.0) | 660 (8.2) | 496 (7.7) | 0.250 |
| Other | 2095 (14.4) | 1040 (12.9) | 1055 (16.3) | <0.001 |

| Supplemental table 6. Incidence rate of mortality according to socioeconomic status in patients who underwent isolated surgical aortic valve surgery | | | | | | | |
| --- | --- | --- | --- | --- | --- | --- | --- |
|  | | **Total number of patients** | **Number of events** | **Person time**  **(years)** | | **IR per 100 person years**  **(95 % CI)** | **Percent event** |
| No of patients |  | |  | |  |  |  |
| All | 14 537 | | 6202 | | 117722 | 5.27 (5.14-5.40) | 42.7 |
| Gender |  | |  | |  |  |  |
| Men | 8059 | | 3120 | | 64082 | 4.87 (4.70-5.04) | 38.7 |
| Women | 6478 | | 3082 | | 53640 | 5.75 (5.54-5.95) | 47.6 |
| Marital status: |  | |  | |  |  |  |
| Married / cohabiting | 8192 | | 3268 | | 68783 | 4.75 (4.59-4.92) | 39.9 |
| Never married | 1711 | | 547 | | 13093 | 4.18 (3.83-4.54) | 32.0 |
| Divorced | 2105 | | 780 | | 16092 | 4.85 (4.51-5.20) | 37.1 |
| Widowed | 2529 | | 1607 | | 19753 | 8.14 (7.74-8.54) | 63.5 |
| Education: |  | |  | |  |  |  |
| <10 years | 6081 | | 3335 | | 51220 | 6.51 (6.29-6.74) | 54.8 |
| 10-12 years | 5523 | | 1962 | | 43656 | 4.49 (4.30-4.70) | 35.5 |
| >12 years | 2769 | | 806 | | 21644 | 3.72 (3.47-3.99) | 29.1 |
| NA | 164 | | 99 | | 1201 | 8.25 (6.70-10.04) | 60.4 |
| Income: |  | |  | |  |  |  |
| Q1 (lowest) | 2908 | | 1837 | | 23482 | 7.82 (7.47-8.19) | 63.2 |
| Q2 | 2908 | | 1535 | | 23972 | 6.40 (6.09-6.73) | 52.8 |
| Q3 | 2907 | | 1322 | | 23935 | 5.52 (5-23-5.83) | 45.5 |
| Q4 | 2907 | | 938 | | 24615 | 3.81 (3.57-4.06) | 32.3 |
| Q5 | 2907 | | 570 | | 21718 | 2.62 (2.41-2.85) | 19.6 |

*IR= Incidence rate. NA= Not analysed. Q = Quintile, Q1= Lowest income level, Q5 = Highest income le

Isolated SAVR between

1997-2020

N=17860

n=17 860

Patients with history of endocarditis
n=1168

Patients without diagnosis of aortic stenosis
n=1532

Final cohort of isolated SAVR
n=14 537

Patients with congenital heart disease
n=623

**Supplemental figure 1.** Flowchart for inclusion and exclusion process.


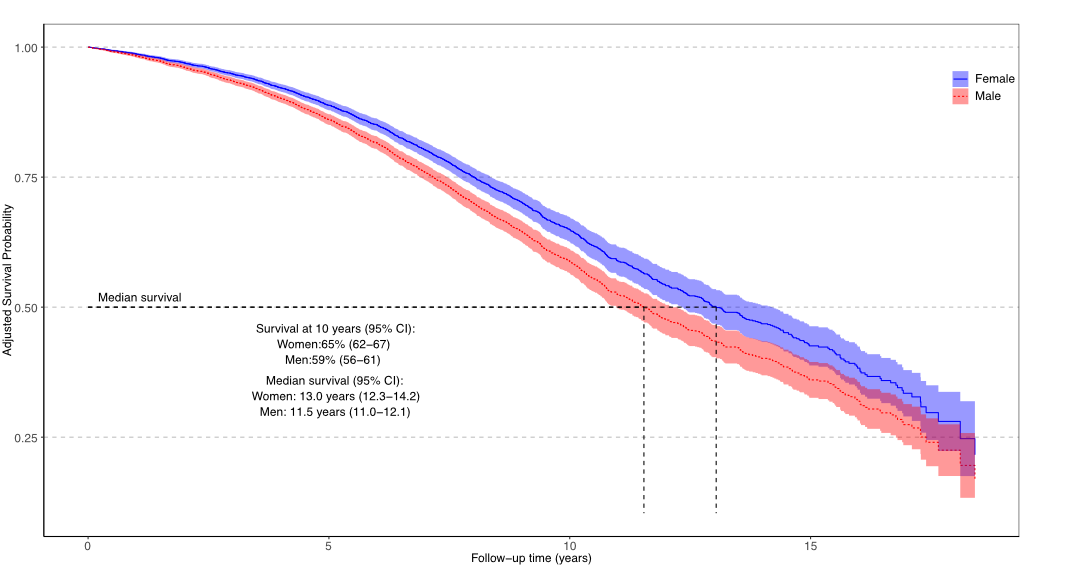


**Supplemental figure** **2**. Adjusted median survival in SAVR patients.


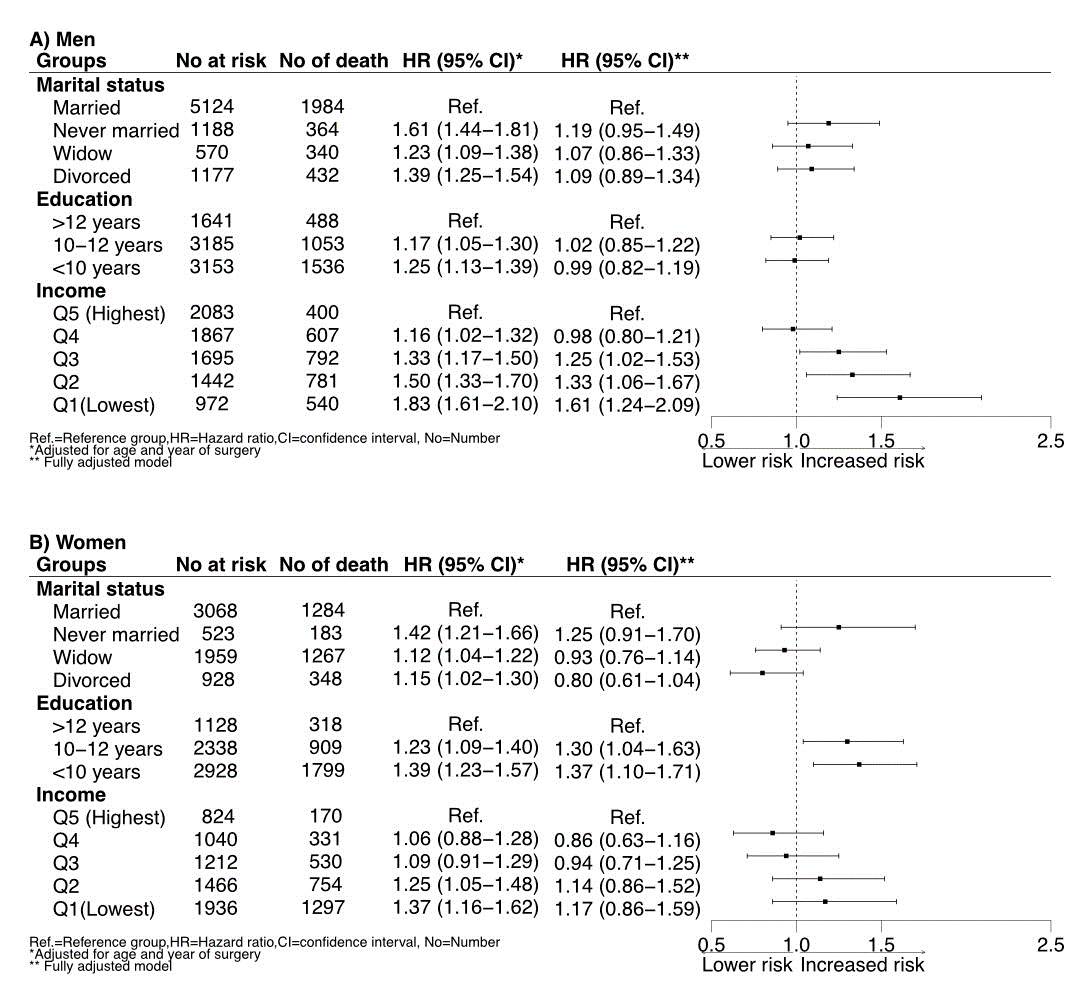


**Supplemental figure 3.** Multi-adjusted Hazard ratio stratified by sex.


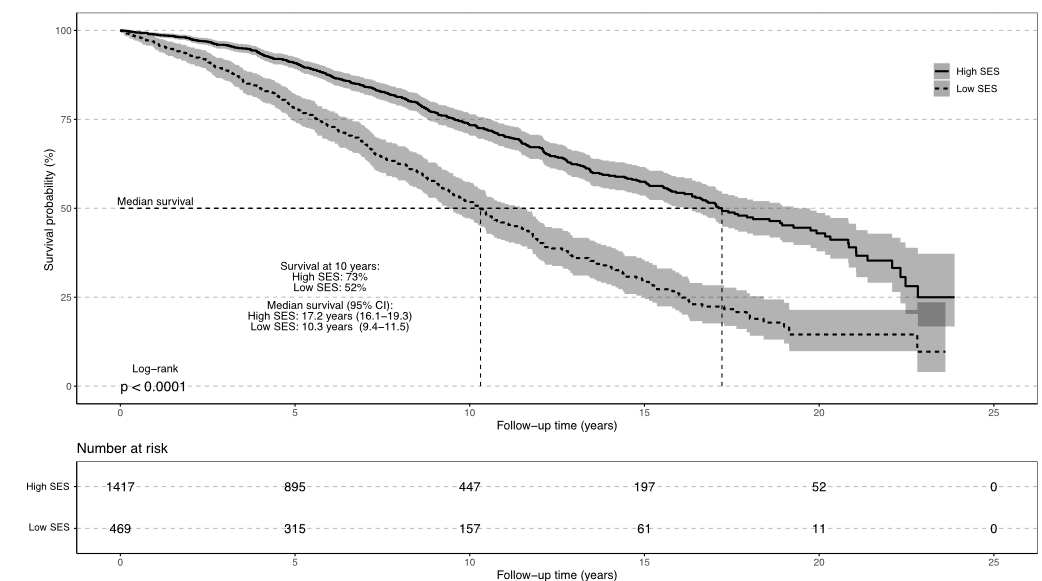
**Supplemental figure 4.** Unadjusted median survival in SAVR patients stratified by SES.
